# Supplementary material for: Are carbon emissions trading and green financial instruments synergistic? -Comprehensive quantitative research based on content analysis
Source: PLoS One. 2024 Mar 7;19(3):e0298601. doi: 10.1371/journal.pone.0298601 (PMC10919601; doi:10.1371/journal.pone.0298601)
Supplement: S1 Data — (ZIP) [file pone.0298601.s001.zip › Data/地级市Z_F工作报告.zip!/─■╧─/╓╨╬└╩╨/└┤╫╘╣1⁄2╓┌║┼í╛┬φ┐╦╩2╛▌═°í┐_2009.docx]

各位代表：

现在，我代表市人民政府向大会作工作报告，请予审议。并请政协委员和列席人员提出意见。

2008年工作回顾

2008年，是极不寻常、极不平凡的一年。我们在自治区党委、政府和市委的坚强领导下，在市人大、市政协的监督、支持下，以邓小平理论和“三个代表”重要思想为指导，团结和带领全市人民，深入贯彻落实科学发展观，紧紧围绕推进发展、改善民生、恢复生态三大工作重点，抢抓机遇，攻坚克难，开拓创新，扎实工作，较好地完成了市二届人大一次会议确定的各项目标任务，中卫跨越式发展迈出了更加坚实的一步！

——地区生产总值实现双突破，gdp和人均gdp分别突破100亿元和1万元大关；

——香山机场建成通航；

——海原新区开工建设；

——建成了美利工业区；

——启动了大南华半阴湿地区水生态造林工程，海原生态恢复有了希望；

——实施农村危房改造工程，14000户农民住上了新房；

——建成南华山职业中专学校，初步实现了普及高中教育目标；

——建立大病救助制度，保证了无钱就医的重病患者得到有效医治；

——开通农村公交，彻底解决了农民出行难、出行安全的问题，农村道路安全事故起数和伤亡人数下降了90%以上；

——办理市长信箱，架起了政府与群众的“连心桥”，进一步树立了市委、政府的威信；

——建立政府“偿贷基金”制度，终结了一直沿袭的把新增的财政收入分散安排到行政支出和项目上的做法，使可用来干事的财力随着财政收入的逐年增加而增多；

——成功举办了中卫奥运圣火传递。

事实充分说明，2008年是中卫经济平稳较快增长、城乡面貌变化巨大的一年，是工业基础设施迅速完善、发展后劲显著增强的一年，是人民群众生活明显改善、和谐中卫建设取得较大进展的一年。一年来，面对区域性竞争日益加剧和国际金融危机带来的严峻形势，我们在竞争中奋进，在危机中调整，在逆境中发展，确保了全市经济社会又好又快发展。

一、经济实力显著提升

国民经济快速增长。预计全年实现地区生产总值118.4亿元，增长13.2%；完成地方财政收入8.8亿元，增长48.2%。其中一般预算收入4.87亿元，增长26.3%；完成全社会固定资产投资80.2亿元，增长21.8%；城镇居民人均可支配收入11600元，增长19%；农民人均纯收入3498元，增长12%。

工业经济平稳运行。预计完成工业增加值38.8亿元，增长19.1%，实现销售收入106亿元，增长35%。其中，规模以上工业增加值完成34.1亿元，增长21.9%。全市新上、技改、扩建（续建）工业项目71个，完成总投资14.5亿元。造纸、酿酒、冶金化工等六大优势特色产业不断壮大，中冶美利纸业年产40万吨涂布白卡纸、科豪陶瓷年产600万平方米的第4条生产线、海鑫集团年产5万吨合成氨、中宁天元锰业集团年产40万吨硫酸一期、宁夏隆基硅公司二期80台直拉单晶炉、华夏特钢公司40万吨硫酸等重点项目相继开工建设并投产，全市工业化率提高到32.7%，工业经济对gdp的贡献率达到43.4%。

特色农业发展加快。预计完成农业增加值26.1亿元，增长7.5%。全市粮食总产量达到52.13万吨，硒砂瓜总产量达到120万吨，马铃薯总产量达到87万吨，设施蔬菜总产量达到41万吨，枸杞干果总产量达到3.8万吨。完成了“三个百万亩”和“六大特色农业基地”建设任务。硒砂瓜、马铃薯、优质饲草种植面积分别达到100.15万亩、105.6万亩和120万亩；种植小茴香12.2万亩，红枣4.2万亩，优质大米17.2万亩；新增高酸苹果10.6万亩，累计达到22.2万亩；新增枸杞4万亩，累计达到25.5万亩；新增设施蔬菜5万亩，累计达到16.9万亩。香山硒砂瓜成为北京奥运会指定产品。实施设施农业提质增效工程，推广高效设施农业模式化种植12万亩。鸡存栏量达447.6万只。新上规模以上农产品加工项目10个，组建各种专业化合作组织27家，培育流通大户20户。

旅游商贸繁荣活跃。以创建中国优秀旅游城市为契机，配套建设了沙坡头东仪门、生态停车场、东大门游客服务中心、沙雕广场、双狮山长城峰燧等旅游项目28个，完成投资8600万元，全年共接待游客116.5万人次，增长5.3%，实现旅游总收入4.86亿元，增长31.9%。完成中卫商业网点规划和迎水桥大物流园区建设规划，改造建设了一批商贸网点，建成世纪朝阳购物广场等商贸服务中心。金融、保险、餐饮、娱乐等快速健康发展，进出口总额达到2310万美元，增长5.2%；预计第三产业实现增加值43.14亿元，增长11.3%。全市社会消费品零售总额预计达到22.72亿元，增长21.1%。

二、发展后劲明显增强

招商引资初见成效。美利工业区水、电、路及供排水等基础设施基本完善，已具备企业入园建厂生产条件。开工建设规划占地面积20平方公里中宁石空工业园和规划占地面积3.5平方公里海原新区工业园。投资20亿元的100万吨捣固焦项目和投资27亿元的5万吨亚氨基二乙腈项目，投资3.5亿元的24万吨高碳铬铁项目，投资4000万元的3000吨绵羊毛分梳项目已开工建设。全市新建、续建（扩建）招商引资项目41个，到位资金22.7亿元，同比增长15.8%。

中小企业活力增强。全面落实扶持中小企业的各项政策措施，出台《中卫市五佳企业十佳个体工商户考核评选办法》、《中卫市中小企业贷款担保管理办法》和《中卫市中小企业担保贷款贴息资金管理办法》，成立了中卫市中小企业贷款担保公司，财政注入500万元，拓宽企业融资渠道，全力促进中小企业快速发展。全市新增中小企业247家，个体工商户6078户，新增注册资金63675.4万元。

各项改革深入推进。在全区率先推行了以下改革：一是建立政府“偿贷基金”制度，使偿还债务有了资金保障，进一步融资有了信用保障，促进发展有了投资保障。二是完成镇乡机构改革。三是推行农业生产设施所有权证制度。四是探索实行农村宅基地流转及房屋产权证制度，为增加农民财产性收入奠定了良好基础。五是实行部门编制动态管理。

三、城乡面貌变化显著

重点项目和基础设施建设进展顺利。中营高速公路中宁至孟家湾段和沙宁滨河北路全线通车。新建市区道路135公里，新修乡镇油路8条186.7公里，新建改建通村等级公路95条431.3公里。市区东、西环路两座立交桥全面完工。拆迁安置2600户，建成3500亩黄河公园、2000亩香山公园；中宁黄河湿地生态园、南河子公园等项目开工建设。全面完成高速公路宣和至鸣沙段两侧各200米，总长53公里，总面积4万亩经济林整地植树工程；完成美利工业区11条道路、总长40公里绿化，植树16万株、面积2760亩，启动实施8000亩绿化带整地及浇水管网工程；完成新区11条道路和市区6公里景观水道绿化，种植草坪125万平方米、各类乔灌木490万株，总面积330万平方米。城市绿化率达到24.5%，城镇化率达到32%。建成各类水利水保及农村安全人饮工程51处，农村6.22万人饮水安全得到保障。

海原新区建设进展顺利。经国务院批准，海原县行政区、工业区搬迁到该县三河镇。海原新区规划用地面积30.7平方公里，3月份完成新区总体规划和控制性详规。共征地1.34万亩，拆迁524户，建成2个农民安置点。完成行政中心办公楼主体工程、恋家大沟回填工程。开工建设城市道路26条54.8公里、南坪水库管网及水厂工程，18家单位办公楼、10栋3万平方米廉租住房和经济适用住房、中小学及教师培训中心、新区医院、宾馆以及供排水等市政工程年内完成。

四、民生民利日益改善

市政府承诺的“十一件实事”基本完成。建成川区193个、山区21个行政村信息终端服务站点。基本建成南华山职业中专学校，秋季招生2043名。开工建设市中心血站、市120急救中心。建设经济适用住房970套和廉租住房92套。开通农村公共交通客运线路，投放农村公交车178辆，农村通公交线路覆盖率由零提高到96.2%。建成3.6万平方米社会福利院工程，完成殡仪馆迁建。开展法律咨询、法律服务3.2万人次。建立市区治安机动巡逻队。建设红梧、杨渠等“塞上农民新居”和新农村示范点46个4598户，改造旧庄点107个，拆除土坯房5682户，完成“平改坡”2570户，新建坡屋顶房3596户。建成香山公园。正在实施东园农贸市场改造。

社会保障覆盖面逐步扩大。建立健全大病医疗救助制度。参加城镇基本养老保险3.43万人、失业保险3.16万人、工伤保险3.1万人、城镇居民基本医疗保险7.85万人、城乡居民享受最低生活保障8.17万人。住房公积金工作成效显著。全市缴存比例全部提高到12%，缴存余额为3.3亿元，贷款余额为1.3亿元。城乡就业工作扎实推进。新增就业岗位8069个，组织劳务输出18.1万人，实现劳务收入9.7亿元。培训下岗失业人员、农民工等1.5万人，城镇登记失业率4.03%。义务教育“两免一补”资金及时到位。共为19.6万名学生免除学杂费6079万元，为17.96万名学生免除教科书费1779.2万元，为1.65万名寄宿生补助生活费833万元。城镇困难群众取暖补贴范围进一步扩大。为纳入城市集中供暖网的低保户每个采暖期发放400元供暖补贴，沙坡头区补贴户数1659户。

五、社会事业全面进步

节能减排和生态建设扎实推进。扩大沙坡头区饮用水水源保护区范围，关闭自备水源21处。实施10家11项重点工业节能减排项目，集中治理11家电石、铁合金生产企业27台矿热炉污染，关停整顿高污染企业4家，取缔非法采矿点26处，整合非煤矿山106处。预计全年万元gdp综合能耗下降4%，二氧化硫排放量和化学需氧量同比分别削减11.2%和10.5%，圆满完成自治区考核指标。规划实施150万亩大南华水生态造林工程，造林20.3万亩，植树1350万棵；完成海原新区凤凰山1100亩绿化工程和腾格里沙漠东南缘43万亩防沙治沙示范项目规划，生态建设迈出实质性步伐。

民主法制和平安中卫建设不断加强。高度重视信访工作，进一步完善市级领导及部门负责人信访接待日制度，全年共受理群众来信来访6670件（次），同比下降5.91%。完善治安巡逻防控机制，加强酒吧等娱乐场所监管，加快公安“三基”工程建设，着力打击刑事犯罪活动，成功破获李小龙涉黑团伙案，社会治安得到了明显好转。扎实开展工矿商贸、道路交通、消防安全和水上交通等专项整治，全年无重特大安全事故发生。党的民族政策深入贯彻，宗教事务管理依法加强。大力推行政务、村务公开，村民自治制度有效坚持。工会、共青团、妇联等工作稳步推进。自觉接受人大法律监督、工作监督和政协民主监督，认真办理人大代表议案、建议和政协委员提案，办结率达到100%。

各项社会事业全面进步。深化科技特派员创业行动，建成农业科技“110”服务站31个，培育信息服务示范点30个。全市“两基”工作顺利通过国家验收，高中教育基本普及，高中阶段入学率达到92.9%。全市高考实现新突破，大专以上录取人数5471人，比上年增加596人，其中本科录取2527人，比上年增加251人。中宁县“教育强县”创建工作通过自治区政府评估验收。广泛开展“全民健身与奥运同行活动”，成功举办奥运火炬传递活动。举办广场文化123场次，海原《大山的女儿》在区、市巡回演出获得圆满成功。新建乡镇文化站6个，市体育馆开工建设。成立岩画文物管理所，岩画等文物和非物质文化遗产得到有效保护。新闻传媒业进一步发展，广播电视覆盖面逐步扩大。建成标准化村卫生室404个、社区卫生服务机构12个，为乡镇卫生院配备救护车27辆，新型农村合作医疗参合率达90.21%。认真落实计划生育少生快富、奖励扶助各项优惠政策，全市人口出生率、自然增长率分别为14.82％。和10.12％。。推行食品药品随机抽检制度，确保了食品药品安全。统计工作水平有新提高，参谋助手作用有效发挥。成功举行地震应急演练，国防教育、人民防空、双拥共建、民兵预备役建设扎实推进。档案、地方志、外事侨务、老龄、残疾人等工作取得新成绩。

六、行政效能显著提高

认真落实党风廉政建设责任制，政府系统惩治和预防腐败体系建设扎实推进，商业贿赂专项治理深入开展，行政用权有效规范。出台效能建设15项制度，组织34个部门（单位）99名处级干部对年度重点业务工作目标进行闭卷考试，对近7000名公职人员进行了培训，各级组织依法行政水平进一步提高。建立市长督办副市长和副市长督办部门重点工作制度，形成一级督办一级、一级对一级负责的执行落实机制。完善市长信箱工作制度，全年共办理群众来信来电922件。全年共召开各类行政工作会议291次，其中：全市经济观摩会2次，政府全体会议3次，政府常务会议44次，市长办公会议61次，专题会议181次，政府科学决策水平不断提高。组建政务服务中心，提供“一站限时”便民服务。规范工作人员公务出差、考勤、人事调配、国有资产管理和公务用车管理，加大对机关事业单位工作人员上岗清查力度，干部作风明显好转。

各位代表：

回顾过去，我们付出了很多艰辛，干了很多大事，实现了很多突破。这些成绩的取得，是自治区党委、政府和市委正确领导的结果，是人大及其常委会、政协和各民主党派监督支持的结果，是全市各族人民辛勤劳动、共同努力的结果。在此，我代表市人民政府，向全市广大工人、农民、知识分子、干部和离退休老同志，向各民主党派、各人民团体和社会各界人士，向驻卫部队、武警官兵和公安干警，向中央、自治区驻卫单位，向所有关心和支持中卫发展的朋友，表示衷心的感谢并致以崇高的敬意！

回顾过去的一年，我们也清醒地认识到，在前进的道路上仍存在很多困难和问题：一是人均创造社会财富少。人均gdp在全国333个地级行政单位中，排位倒数第61位，低于全国平均水平53.9%。二是工业化水平还处于较低层次。工业产值小、结构不尽合理、技术创新薄弱、产品附加值低。特别是进入10月份以来，受金融危机影响，工业生产明显回落。三是农业生产受到人均耕地资源少、自然条件差、技术进步慢等制约，农民增收难度大，移民任务十分繁重。四是财政收支平衡压力大。今后3年，是偿还银行债务任务最重的3年，也是偿贷基金起步建立、积累资金能力最弱的3年，一重一弱加重了财政收支平衡的压力。五是受全国经济形势影响，招商引资困难加大。六是个别公务员责任意识淡薄，工作作风漂浮。对这些困难和问题，我们要始终保持清醒头脑，增强忧患意识，以对党对人民高度负责的使命感和责任感，更加兢兢业业，扎扎实实地做好政府各项工作。

2009年目标任务

2009年，严峻挑战与重大机遇并存。面对“明年可能是新世纪以来我国经济发展最为困难的一年，也是蕴含重大机遇的一年。”这样一个特殊而关键的时期，只有抓住机遇，才能化解危机战胜挑战，只有战胜挑战，经济增长才能进入一个新的发展阶段。为此，我们要以更加明晰的工作思路，更加得力的工作措施，更加扎实的工作作风，奋力推进中卫经济社会跨越式发展。

中卫跨越式发展的总体构想是：突出“三大定位”，围绕“一大目标”，落实“五大任务”。三大定位是：把中卫建成生态旅游与交通枢纽城市，枸杞硒砂瓜马铃薯之乡，精细化工与新材料工业基地。一大目标是：到2020年，全市人均地区生产总值比2000年翻三番，超过3万元，与全国同步迈入更高水平的小康社会。五大任务是：一是加大以工促农、以城带乡、以川济山力度，大力推进社会主义新农村建设；二是切实转变经济发展方式，加快提升工业生产能力；三是着力解决好民生问题，积极构建社会主义和谐中卫；四是全面加强城市生态建设，把中卫建成沿黄城市带中最具特色美的城市；五是全面推进以水生态为中心的可持续发展战略，再造中卫秀美山川。

2009年工作的总体思路是：全面贯彻落实党的十七大、十七届三中全会，自治区党委十届七次全体（扩大）会议、自治区经济工作会议和市委二届五次全体（扩大）会议精神，深入贯彻落实科学发展观，进一步解放思想、抢抓机遇、苦干实干，着力推进新型工业化、农业现代化、城乡一体化、旅游特色化进程，着力改善民生和促进社会和谐，为实现中卫经济社会跨越式发展奠定坚实基础。

2009年经济社会发展的主要预期目标是：地区生产总值130亿元，增长11%；地方财政一般预算收入5.7亿元，增长15.5%；全社会固定资产投资91亿元，增长16.7%；规模以上工业增加值39亿元，增长15%；城镇居民人均可支配收入12700元，增长9%；农民人均纯收入3800元，增长8%；全市社会消费品零售总额达到26亿元，同比增长14.6%；城镇登记失业率控制在4.2%以内；人口自然增长率控制在12％。以内；单位生产总值能耗降低4个百分点，化学需氧量、二氧化硫排放量分别下降10%。

实现上述目标，我们要更加重视跑项目争资金，进一步改善城乡生产生活基础设施条件；更加重视广招商大引资，进一步提高工业生产能力；更加重视调结构提质量，进一步促进农业增产增收；更加重视解难题办实事，进一步提高人民群众生活水平；更加重视多投入促和谐，实现全市经济社会又好又快发展。

重点实施九个“强力推进”：

一、广招商大引资，强力推进工业发展

狠抓重点招商项目。着力促进十大工业项目开工，力争引进投资5000万元以上项目10个，全市招商引资额达到25亿元。重点抓好中冶美利纸业二期高档文化用纸，湖北宜化集团煤化工、盐化工，江苏扬农集团精细化工，中电投2×330mw和中意生物热电联产，江苏泰兴锦汇化工公司1万吨燃料中间体，浙江嘉化公司8万吨双氧水，温州山联集团5万吨特种纸，中宁隆基公司二期2800吨单晶硅，华夏特钢30万吨镍铁，湖南金德24万吨铬铁等重点招商项目开工建设。抓好永安公司100万吨捣固焦项目一期、昊丰伟业公司循环经济产业项目一期、中宁天元公司7.2万吨金属锰等在建项目，争取年内投产。建成1家产值超过20亿元、2家产值超过10亿元、5家产值超过5亿元的骨干企业，规模以上企业达到85家，确保全年完成工业增加值43亿元，增长15%，实现销售收入126.5亿元，增长15%，其中规模以上工业增加值39亿元，实现销售收入110亿元，分别增长15%。

培育主导产业。依托本地资源优势，壮大造纸业，做强冶金化工业，培育新型建材业，改造传统机械制造业，提升农副产品加工业。加大中冶美利纸业、昊丰伟业、宁夏红集团、大河机床、隆基硅公司、天元金属锰、科豪公司等骨干企业的支持力度，在用地、税收等方面向骨干企业倾斜。同时，进一步完善和落实推动全民创业、扶持中小企业发展的各项政策措施，鼓励和支持中小企业二次创业，引导本地资金与外来资本嫁接配套，积极帮助中小企业拓宽市场，提高效益。进一步优化发展环境，全面落实各项激励政策，降低行政性收费，促进中小企业健康快速发展。

二、调结构提质量，强力推进农业增产农民增收

大力发展现代农业。继续大力实施“三个百万亩”工程，从数量扩张向提质增效转变。硒砂瓜、马铃薯、优质饲草种植面积分别达到100万亩、110万亩和120万亩。巩固提高六个种植基地，种植枸杞29万亩，新增3.5万亩；种植小茴香12万亩；种植红枣32.4万亩，新增10万亩；种植高酸苹果26万亩，新增3.8万亩；种植优质米17万亩；新建日光温室2.5万亩（其中海原新增2.3万亩），全市累计达到20万亩。加快发展清真牛羊肉产业，稳定猪产业，恢复鸡产业。探索发展沙漠节水农业和生态观光农业。重视农副产品加工和销售，新组建各类农产品流通组织和专业协会15家，新上技改、扩建规模以上农产品加工项目10个。进一步拓宽农民外出务工渠道，提高政府组织化程度，充分发挥南华山职业中专学校平台作用，分期分批开展劳动技能培训，努力增加农民工工资收入，确保实现农业增产农民增收目标。

改善农村环境。加强农业基础设施建设，实施农业综合开发、基本农田整治和标准农田示范区项目。抓好灌区沟渠整治和山区引水补灌工程，增强农业防灾避灾能力。加强农村基础设施建设，积极推广使用沼气、太阳能等清洁能源，改善农村卫生环境。新建“塞上农民新居”示范点10个，突出抓好各类公路沿线村庄环境整治。加快实施生态移民和危窑危房改造工程，完成生态移民新村6个，改造危窑危房5000户。

促进山川协调。加快推进“以川济山，山川共济”战略，抓好山川优势特色产业对接、劳务市场对接、产业扶贫对接，认真落实扶贫开发的各项措施，大力开展项目扶贫、产业扶贫和市直单位定点帮扶，形成社会帮扶长效机制，整合各类扶贫项目资金，加快实施生态移民项目，从根本上解决山区群众生活出路问题。

三、高起点大投入，强力推进城镇化进程

切实维护城乡规划。认真贯彻《城乡规划法》，充分发挥规划的龙头作用，进一步完善城乡总体规划，配套编制控制性详细规划和区域性专项规划。修编土地利用总体规划，调整优化土地利用结构和布局。加大城乡规划执行力度，严肃查处违法占地、违规建设等行为，切实维护城乡规划的统一性和权威性。

加快城市建设步伐。重点实施八项工程：一是建成美利森林公园；二是完成腾格里湖基础设施工程；三是完成应理生态房地产开发项目；四是建成美利工业区污水处理厂；五是建成滨河南路，机场路延伸，美利路、文远路、体育路、平安路等49.5公里城市道路，完成新区中山街、文昌街、长城街、应理北街和蔡桥路“四街一路”改造工程；六是完成市区道路绿化改造工程；七是完成新区供热站扩容，环城东路、公园路中水管网配套和医疗垃圾无害化处理等工程；八是起步“五创”工作，即创建国家卫生城市、国家园林城市、国家环境保护模范城市、全国文明城市和中国优秀旅游城市。

加快建设海原新区。启动海原新区行政中心广场建设、苋麻河治理、主干道绿化等项目，年内完成学校、医院一期工程及54.8公里城市道路，10栋3万平方米廉租住房及经济适用住房，110kv变电所等公共服务设施建设。确保明年5月底，道路、供排水、电力、通讯、行政机关办公大楼等一期工程投入使用。建成25家单位办公楼，力争到2009年底，大部分部门（单位）都能进入新区办公。

推进城乡一体化进程。加大中宁老城改造，启动新区建设，完善公共设施，提升城市品位，辐射带动县域经济发展。改造海原老城，确保繁荣稳定。加快中心集镇电网、路网、供排水、垃圾处理等服务设施配套，形成以市区为中心、县城为重点、小城镇为主体的多元格局，促进城乡协调发展。城镇化率达到39%。

四、活商贸促旅游，强力推进第三产业发展

壮大旅游产业。开发景区景点，实施腾格里沙漠湿地开发、沙坡头基础设施配套、大漠景区整体向北延伸开发等项目，整合精品旅游线路，挖掘旅游文化内涵，办好大型旅游节庆活动，提升旅游文化品位。大力整顿旅游文化市场，规范旅游行业管理。加强区域联合，促进我市与周边城市旅游资源共享。打响沙坡头5a级旅游区品牌，加强旅游促销，开拓客源市场。全年接待游客118万人次，实现旅游总收入5亿元，增长3%。

培植房地产业。精心组织香山公园东侧商品住宅开发项目。建成黄河花园二期、美利城市风景、水木兰亭等房地产开发工程；开工建设美利东方明珠、瑞丰家园、山水众一、正丰·香格里等住宅项目，全力推动房地产业健康快速发展。

提升服务产业。进一步完善城市商业网点和专业市场布局，扎实推进“万村千乡”市场工程。加强与国内外大型商贸和物流公司合作，积极发展新型物流业态。开工建设迎水桥大物流园区。发展路铁联运，加快航空、陆路和电子口岸建设。培育壮大文化信息、体育健身、金融保险、医疗保健、餐饮娱乐等产业。进一步加强市场监管，规范企业经营行为，营造安全、有序的市场交易环境。

五、解难题办实事，强力推进民生事业

重点落实三大社会保障措施，集中办好15件实事。

一是落实劳动就业保障。加快构建城乡一体的就业服务体系，拓展就业渠道，新增就业岗位8000个。落实培育50名青年创业带头人实施方案，办好50名青年创业者电视大赛，让更多的人积极投身于全民创业行动之中，形成“百姓创家业、企业创新业、能人创大业、干部创事业”的自主创业、充分就业的浓厚氛围。

二是落实医疗卫生保障。财政安排医疗卫生支出4596万元，增长13.8%，加强乡镇卫生院、村卫生室建设，努力改善卫生院基础设施不足、医疗设备落后和人才缺乏状况。全面推行城镇居民医疗保险工作，城镇居民医疗保险覆盖率达85%以上；实行新型农村合作医疗门诊、住院“双统筹”，全市农民参合率达到91%以上。进一步完善大病医疗救助制度，着力解决困难群众看病难、看病贵问题。

三是落实养老失业保障。抓好社保基金扩面征缴工作，做好企业离退休人员养老待遇调整工作，确保全市所有企业离退休职工养老金按时足额发放。认真落实农村低保制度，逐步扩大保障覆盖面，确保低保对象和五保户“应保尽保”。建设廉租住房120套，发放廉租住房租赁补贴145.8万元。落实村干部养老保险制度。积极发展社会福利事业和慈善事业。

四是为民办好15件实事：

⑴建立农村养老保险制度。为沙坡头区男60周岁、女55周岁以上农民每人每月发放20元养老补贴，并以此为基础，加快建立农村养老保险制度。⑵实施“塞上农民新居”和农村危房改造安居工程。完成沙坡头区2万户土坯房改造，让10万人迁入新居。⑶为农民免费健康体检。为沙坡头区45岁以上7.13万农民群众免费进行肝功、血糖、血压等健康项目体检。⑷增加农村电视收视频道。沙坡头区川区电视节目由8套增加到26套，山区由4套增加到42套。⑸实施卫宁灌区3万亩盐渍化土地改造工程。⑹建设中卫市蔬菜专业批发市场。⑺建设“两馆一中心”三大公共建筑。建成建筑面积1万平方米、4000座的综合性体育运动馆；建成博物、图书、文化为一体的建筑面积1.5万平方米的博物馆主体工程；建成游泳、健身、羽毛球、壁球及各类训练为一体的建筑面积2.3万平方米的文体娱乐中心主体工程。⑻开展“住房年”活动。建设经济适用住房8万平方米，发放廉租住房租赁补贴；提高住房贷款额度，新发放住房公积金个人贷款6500万元。⑼实施天然气入户工程。新增用气居民5000户，新建汽车加气站一座，敷设美利工业区通气高压管线。⑽完善城市公共卫生设施。新建垃圾收集站1处、公厕20处，设置大垃圾箱20个、果皮箱750个，进一步美化、净化城市环境。⑾改造城中村。对文昌镇、滨河镇共5个村危旧平房进行拆迁改造，改善老城区环境。⑿让老年人残疾人免费享受公共服务。⒀全面消除沙坡头区中小学危房。⒁完成行政村村村通油路任务。建设通村油路196公里，实现沙坡头区村村通油路目标。⒂开展全民创业活动。改造东园农贸市场，建成全民创业城；培育50名创业领军人。全年培育200个小老板、200个小企业，提供2000个就业岗位。

六、跑项目争资金，强力推进项目建设

抢抓国务院《关于进一步促进宁夏经济社会发展的若干意见》和《关于进一步扩大内需促进经济增长十项措施》两大机遇，成立“争取重点项目办事机构”，派专人驻京，重点抓好47个项目的争取工作。工业方面重点争取8个项目。主要是：中冶美利集团林纸一体化15万吨高档印刷纸、30万吨杨木化机浆，湖北宜化30万吨硝铵，昊丰公司5000万块蒸养砖，大河风电机座偏轴加工研发等项目。农田水利、农村基础设施方面重点争取7个项目。主要是：沙坡头灌区续建配套与节水改造、兴仁农村饮水安全、黄河卫宁段治理、农村卫生基础设施等项目。交通、城市基础设施方面重点争取13个项目。主要是：国家和自治区对香山机场补贴、火车站迁建、农村公路改造、新区基础设施建设、重点流域污染治理、老城工业区治理等项目。农业、生态建设方面重点争取7个项目。主要是：大南华1000平方公里水生态造林、腾格里沙漠东南缘防沙治沙示范工程、生态移民等项目。商贸旅游方面重点争取4个项目。主要是：迎水桥大物流园区、沿黄生态恢复及湿地保护、腾格里沙漠湿地生态旅游等项目。社会事业和改善民生方面重点争取8个项目。主要是：职业学校和农村中小学改造、农村危房改造安居工程、农民养老保险等项目。

七、建机制理体制，强力推进改革创新

加大企业改制力度。认真研究解决改制企业遗留问题，加快分离社会办企业职能，加快推进企业改革、改组、改制，进一步优化要素，激活存量，提升效益。全面完成中卫宾馆、市种子公司、设计院和水务局下属6个实体的改制任务。

继续深化农村综合改革。深入推进粮食流通体制、水管体制、集体林权制度改革。努力探索工业反哺农业、城市支援农村、财政支持“三农”的长效机制，积极化解村级债务。

不断深化财税体制改革。加强非税收入管理，扩大偿贷基金规模，提高财政保障能力。严格部门经费预算管理，实行部门办公用品集中定点采购，降低行政运行成本。规范转移支付激励机制，缓解县乡财政困难。

多层次构建融资平台。抢抓国家实施适度宽松的货币政策机遇，持续加大对大项目的信贷支持，全力支持“三农”、中小企业等资金需求，支持出口企业稳步发展。充分发挥中小企业信用担保作用，着力培育中小企业集群，引导支持全民创业。

八、多投入促和谐，强力推进社会进步

优先发展教育。积极开展教育强县（区）创建工作，普及高中阶段教育和学前教育，关注特殊教育。切实保障义务教育经费“三个增长”，持续改善办学条件。大力发展职业教育，加快南华山职业中专学校后续工程和配套设施建设，开工建设中宁职教中心和实训基地，办好农村职业高中远程教育，确保高中阶段入学率达到93%，职业教育与普通高中比例大体相当。继续深化教育教学改革，切实加强教师队伍建设，全面提高教育教学质量。

发展科技事业。大力推进中卫可持续发展实验区建设，加快实施专利战略、品牌战略，组织实施一批科技计划项目，培育骨干企业进入自治区高新技术行列。建立充分调动科技人员积极性的新机制，开展好科技下乡和科普教育活动。实施人才强市战略，培养和引进各类高层次人才和急需人才。积极筹建科技信息中心，争取在科技阵地建设上有新突破。

加强文化建设。充分挖掘中卫文化底蕴，全力打造“文化中卫”名城。广泛开展全民健身活动，不断提高竞技体育水平。加大岩画、古长城等文物保护和非物质文化遗产保护工作，挖掘整理地方民俗文化。丰富文化产品，广泛开展广场文化活动。加强网络文化建设和管理，营造良好网络环境。重视文艺精品创作，加强文艺人才培养，提升特色文化。深入开展群众性精神文明创建活动，不断丰富城乡群众精神文化生活。

加大生态环境保护力度。加快实施150万亩大南华水生态造林工程，2009年造林30万亩。完成8000亩美利工业区绿化工程和43万亩腾格里沙漠东南缘防沙治沙工程立项。

推进节能减排。围绕重点行业、重点领域和重点企业，突出工业、建筑、交通和机关节能，加强对重点企业监管，着力实施一批节能工程和节能项目，下决心淘汰落后产能。认真抓好节水示范区和美利工业区循环经济示范区建设，确保沙坡头区污水回用项目正常运转。切实加强对重点区域水、空气、噪音污染防治，全面完成节能减排目标。

加强人口和计划生育工作。进一步完善利益导向机制，加强计划生育基础设施建设，继续实施“少生快富”工程，全面落实各类奖励扶助政策，稳定低生育水平，切实提高出生人口素质，实现人口与经济、社会、资源、环境协调可持续发展。

促进社会和谐稳定。全面落实各级安全生产责任制，加强对重点行业与领域的安全隐患排查和专项整治，依法取缔非法采矿行为，严防各类重特大事故发生。坚持食品药品随机抽检制度，依法查处问题食品、药品，保障人民群众健康安全。重视做好群众来信来访工作，加强人民调解和法律援助，加大矛盾纠纷排查化解力度，有效预防和妥善处置群体性事件。继续深入推进平安创建，加强公安信息化和执法规范化建设，构建和谐警民关系；依法严厉打击各类刑事犯罪，确保群众人身财产安全。贯彻民族宗教政策，依法管理宗教事务，促进民族团结进步。加强公民教育、未成年人思想教育和社会公德、职业道德、家庭美德建设。增强全民国防观念，加强国防动员建设，创建双拥模范城。高度重视防震减灾和应急演练工作。进一步做好统计、人防、广播电视、新闻出版、外事侨务、气象、市志、工会、共青团、妇联、老龄、残联等工作，促进社会各项事业协调发展。

九、提效能树新风，强力推进民主法制建设

坚持依法行政。深入开展“五五普法”。认真贯彻落实《全面推进依法行政实施纲要》、《行政许可法》和《公务员法》，强化行政执法责任制和行政问责制。自觉接受人大及其常委会的法律监督、政协的民主监督和人民群众的监督，确保权力在阳光下运行。认真办理人大代表议案、建议和政协委员提案。诚恳听取民主党派、工商联、无党派人士及各人民团体意见，建立健全社情民意反映机制和重大事项社会公示、听证制度。健全基层群众自治机制，保障人民群众享有更多更切实的民主权利。

提高行政效能。进一步转变思想作风、领导作风和工作作风，建立健全抓落实的工作机制，完善目标责任考核体系，强化政务督察、效能监察和绩效评价，坚决杜绝推诿扯皮、各行其是现象，确保政令畅通。抓好政府组成部门参加政府重要议事活动评议工作，提高决策质量和效率。办好市长信箱和政府网站，及时解决群众诉求。建设乡镇便民服务中心，为农民群众提供“一站式”限时服务。广泛深入基层，开展调查研究，坚持不懈地为群众办实事、解难事、做好事。

树立廉政新风。认真贯彻落实党风廉政建设责任制，扎实推进政府廉政建设。积极构建完善、有效的廉政监督机制，严格执行廉洁自律各项规定，做到用制度管权、按制度办事、靠制度管人，从源头上预防和治理腐败。牢记“两个务必”，坚持量力而行，勤俭办事，坚决反对和制止奢侈浪费。开展机关作风民主评议活动，坚决纠正损害群众利益的不正之风，努力塑造政府新形象。

各位代表：

回顾2008年成绩与进步，令人鼓舞；展望2009年发展与未来，催人奋进。让我们在自治区党委、政府和市委的正确领导下，始终保持蓬勃向上的朝气，始终保持奋发有为的状态，始终保持干事创业的激情，变压力为动力，化挑战为机遇，坚定发展信心，狠抓工作落实，奋力开创中卫跨越式发展的新局面！
